# Seroepidemiology of *Rickettsia conorii* in dogs in Portugal: a comprehensive 12-year retrospective study (2013–2024)

**DOI:** 10.1186/s13071-025-06859-z

**Published:** 2025-06-23

**Authors:** Ricardo Lopes, Hugo Lima de Carvalho, Andreia Garcês, Cátia Fernandes, Ana Patrícia Lopes, Ângela Martins, Elsa Leclerc Duarte, Luís Cardoso, Ana Cláudia Coelho

**Affiliations:** 1 Department of Veterinary Sciences, University of Trás-os-Montes e Alto Douro (UTAD), Vila Real, Portugal; 2 Department of Veterinary and Animal Sciences, University Institute of Health Sciences (IUCS), CESPU, Gandra, Portugal; 3 CEDIVET Veterinary Laboratories, Lionesa Business Hub, Leça do Balio, Portugal; 4 Wildlife Rehabilitation Centre (CRAS), Veterinary Teaching Hospital, UTAD, Vila Real, Portugal; 5 Animal and Veterinary Research Centre (CECAV), Associate Laboratory for Animal and Veterinary Sciences (AL4AnimalS), UTAD, Vila Real, Portugal; 6 Anicura Santa Marinha Veterinary Hospital, Vila Nova de Gaia, Portugal; 7 Department of Veterinary Medicine, School of Science and Technology, University of Évora, Évora, Portugal; 8 Mediterranean Institute for Agriculture, Environment and Development (MED), Global Change and Sustainability Institute (CHANGE), University of Évora, Évora, Portugal

**Keywords:** Canine seropositivity, One health, *Rickettsia conorii*, Seroprevalence, Tick-borne infections, Vector-borne diseases, Zoonosis

## Abstract

**Background:**

Mediterranean spotted fever (MSF), caused by *Rickettsia conorii*, is a zoonotic tick-borne disease of important public health concern, particularly in the Mediterranean Basin. Dogs serve as key sentinels for MSF due to their exposure to vector ticks and close contact with humans. To date, no comprehensive study in Portugal has investigated epidemiological risk factors in dogs infected with or exposed to *R. conorii*.

**Methods:**

Seropositivity to *R. conorii* was determined using an immunofluorescence antibody test (IFAT), with titres categorised as negative, low positive, moderate positive and high positive. Statistical analyses included the chi-squared test and univariable logistic regression to assess associations between seropositivity and geographical region, season, month, size, breed, sex and age.

**Results:**

This study analysed 2457 canine samples submitted from 228 veterinary medical centres across mainland Portugal and insular autonomous regions between 2013 and 2024. The overall seroprevalence of *R. conorii* was 27.0% (95% confidence interval [CI] 25.3–28.8). Significant differences in seroprevalence were observed amongst regions, with the highest values in the Algarve (48.0%; odds ratio [OR] 3.1, 95% CI 2.2–4.4, *P* < 0.001), Alentejo (35.0%; OR 1.8, 95% CI 0.7−4.6, *P* = 0.210) and Centre (33.8%; OR 1.7, 95% CI 1.4–2.1, *P* < 0.001) regions. Giant breeds had the highest seroprevalence (33.0%; OR 3.0, 95% CI 1.3–6.6, *P* = 0.008), with Irish Setter and Miniature Schnauzer identified as high-risk breeds (75.0%; OR 27.0, 95% CI 1.3–578.4, *P* = 0.035). Male dogs had higher seroprevalence (28.9%; OR 1.2, 95% CI 1.0–1.5, *P* = 0.023), whilst geriatric dogs showed the highest risk of exposure to or infection with *R. conorii* (34.6%; OR 5.2, 95% CI 2.8–9.8, *P* < 0.001).

**Conclusions:**

This study represents the most extensive epidemiological analysis of canine MSF in Portugal. The findings highlight associations between *R. conorii* seropositivity and geographical region, size, breed, sex and age of dogs, advancing the limited knowledge on the epidemiology of *R. conorii* in Portugal and underscoring the need for regional surveillance and targeted prevention to reduce infection risks in both canine populations and public health contexts.

**Graphical Abstract:**

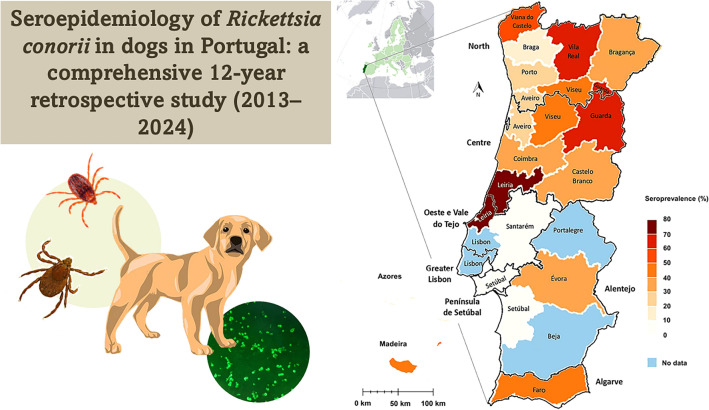

## Background

*Rickettsia* is a genus of obligate intracellular, Gram-negative bacteria belonging to the family Rickettsiaceae. These bacteria are primarily transmitted to humans and animals by vector arthropods, including ticks, lice, mites and fleas [1–3]. *Rickettsia* spp. are classified into two primary groups on the basis of their disease-causing properties: spotted fever group (SFG) and typhus group [4–9].

In recent years, there has been increasing attention to canine vector-borne diseases (CVBD), including those caused by *Rickettsia* spp., due to their increasing prevalence worldwide and also zoonotic potential [10–17]. Factors such as climate change, globalisation, human expansion and an increase of canine populations have contributed to a shifting epidemiology of these diseases, especially those whose agents are transmitted by tick vectors *Rhipicephalus sanguineus* s.l. Amongst the various tick species potentially involved in transmission, *Rh. sanguineus* s.l. is considered the primary vector of *R. conorii* in the Mediterranean Basin, due to its ecological association with dogs and established vector competence [4, 18–22].

Studies worldwide have highlighted the role of shelter dogs as effective sentinels for CVBD due to their increased exposure to vector ticks in overcrowded and poorly controlled environments [15, 17, 23–25]. Serological surveys of owned and shelter dogs in endemic regions have consistently shown high seroprevalence values, reflecting regional differences in tick activity, host exposure and environmental conditions [26–28].

*Rickettsia conorii*, the causative agent of Mediterranean spotted fever (MSF), also known as boutonneuse fever, poses a considerable threat in the Mediterranean Basin and subtropical regions, including southern Europe and North Africa. Dogs, due to their frequent infestation by *Rh. sanguineus* s.l. ticks and close contact with humans, may serve as valuable sentinels for *Rickettsia conorii* infection. Monitoring their seropositivity provides valuable insights into the potential human exposure risk [28–34].

This study aims at analysing epidemiological parameters potentially related to the agent of MSF in dogs in Portugal. It provides an update on the canine seroprevalence of infection with or exposure to *R. conorii* in this country, over 12 years (2013–2024), with the aim of offering valuable insights for both veterinary and public health contexts, and marking the first extensive work of its kind conducted in Portugal.

## Methods

### Sampling, data collection and diagnostic procedures

Blood or serum samples from dogs suspected of tick-borne infections were submitted to CEDIVET Veterinary Laboratories (Portugal). These samples (*n* = 2457) were collected in 228 veterinary medical centres, including clinics and hospitals, across mainland Portugal and the insular autonomous regions. Each sample included a laboratory requisition with the relevant signalment information, such as breed, sex and age, but all data were anonymised. Only samples with complete signalment data – breed, sex and age – were eligible for inclusion. However, animals lacking age information (16.5% of the dataset) were retained in the overall analysis but excluded from age-stratified comparisons. Mixed-breed dogs were excluded from breed- and size-specific analyses. All samples were originally selected on the basis of clinical suspicion of tick-borne disease, as reported by the submitting veterinary medical centres. Breeds were categorised into five size groups: (a) toy (< 5 kg), (b) small (6–10 kg), (c) medium (11–25 kg), (d) large (26–40 kg) and (e) giant (41 kg or more) [35]. Age of the animals was categorised into five groups: (a) puppy, < 1 year old; (b) young, 1 to < 2 years old; (c) adult, 2 to < 6 years old; (d) senior, 6 to < 11 years old; and (e) geriatric, ≥ 11 years old [24, 36].

The serum samples were tested by an immunofluorescence antibody test (IFAT) using commercial slides (MegaFLUO^®^ RICKETTSIA conorii, MEGACOR Diagnostik GmbH, Hoerbranz, Austria) for the detection of immunoglobulin (Ig)G antibodies specific to *R. conorii*, according to the manufacturer’s instructions. Sera were tested starting at a 1:64 dilution, and the corresponding 64 titre (i.e. the reciprocal of that dilution) was established as the cut-off value for determining seropositivity [26, 37, 38]. If a positive result was detected at this dilution, further serial dilutions were performed until a negative result was observed. In this study, those additional dilutions included 1:128, 1:256, 1:512, 1:1024 and 1:2048 [26, 38–40].

Interpretation of antibody titres was categorised as follows:

**Negative/low titre:** results below 64 were classified as negative or indicative of a null or low antibody level, suggesting no exposure to or infection with *R. conorii*.

**Borderline/low positive:** a titre of 64 was considered borderline or low positive, indicating possible exposure, but not necessarily an active infection.

**Moderate positive:** titres of 128 and 256 reflected moderate antibody levels, suggesting likely exposure to and infection with *R. conorii*.

**High positive:** titres of 512 or higher were classified as high, indicative of a strong immune response and likely recent or ongoing infection.

These classifications provide a framework for assessing the degree of exposure and immune response to *R. conorii*, aiding in the interpretation of serological results.

### Statistical analysis

The sample parameters were categorised as: geographical region (NUTS 2: Nomenclature of Territorial Units for Statistics), season, month, size, breed, sex, age and antibody titres. All the data were available in digital format in Sislab^®^ (Glintt, Global Intelligent Technologies, Lisbon, Portugal) and transferred to Microsoft Excel^®^ (Microsoft, Redmond, WA, USA) sheets. Statistical analysis was conducted using the JMP^®^, version 14.3 (SAS Institute, Cary, NC, USA), DATAtab^®^ (Online Statistics Calculator, DATAtab e.U., Graz, Austria) and MedCalc^®^ statistical software version 20.006 (MedCalc Software Ltd, Ostend, Belgium). The chi-squared test was used to compare proportions of positivity, with exact binomial 95% confidence intervals (CI) being established. Univariable logistic regression was applied to identify potential risk factors, that is, odds ratios (OR) of *R. conorii* seropositivity amongst variables/categories. A *P*-value ≤ 0.050 was considered as statistically significant [41].

## Results

### General seropositivity, geographical distribution and seasons

Of the total of 2457 animals included in this study, 664 (27.0%) tested positive, whilst 1793 (73.0%) tested negative to *R. conorii*. The distribution of seropositivity across serological titres is detailed in the Table 1.

**Table 1:** Seropositivity to *Rickettsia conorii* by titre in 2457 dogs

Titre	*n*	%	95% CI
< 64 (negative)	1793	73.0	71.2–74.7
64 (low positive)	317	12.9	11.6–14.3
128 (moderate positive)	131	5.3	4.5–6.3
256 (moderate positive)	86	3.5	2.8–4.3
512 (high positive)	78	3.2	2.6–3.9
1024 (high positive)	49	2.0	1.5–2.6
2048 (high positive)	3	0.1	0.0–0.4

Sera were tested starting at a 1:64 dilution, with a titre of 64 defined as the threshold for seropositivity (titres are expressed as the reciprocal of the corresponding dilutions)

*CI* confidence interval

**Figure 1:**
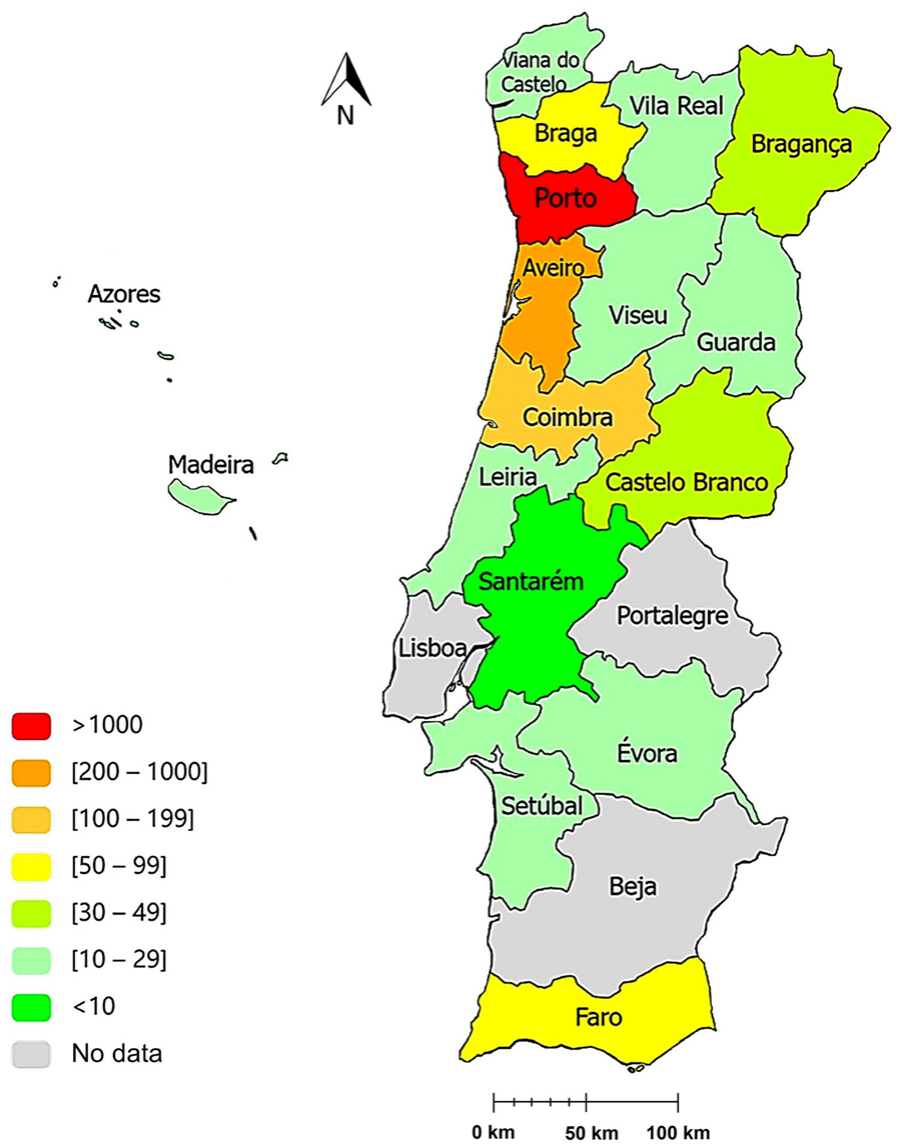
Spatial distribution by the different districts (*n* = 18) of mainland Portugal and insular autonomous regions (*n* = 2) of the 2457 animals included in this study (map drawn in: www.paintmaps.com; accessed on 19 January 2025)

The majority of samples originated from the districts of Porto (*n* = 1393; 56.7%), Aveiro (*n* = 250; 10.2%), Coimbra (*n* = 241; 9.8%) and Faro (*n* = 148; 6.0%) (Fig. 1).

The distribution of results by geographical regions (NUTS 2) of mainland Portugal and insular autonomous regions is presented in Table 2. Data from the Greater Lisbon region were not available for the present study. The seroprevalence of exposure to or infection with *R. conorii* in this study across the different NUTS 2 ranged from 0.0% to 48.0%. The regions with the highest seroprevalence of *R. conorii* were the Algarve (48.0%), followed by the Alentejo (35.0%) and Centre regions (33.8%). The chi-squared test revealed a statistically significant association between region (NUTS 2) and seroprevalence of *R. conorii* (*χ*^2^ = 66.2, *df* = 7, *P* < 0.001). The Algarve presented the highest risk (OR 3.1, 95% CI 2.2–4.4, *P* < 0.001), followed by the Centre region (OR 1.7, 95% CI 1.4–2.1, *P* < 0.001). The North region served as the reference category (23.0%; OR 1).

**Table 2:** Seropositivity to *Rickettsia conorii* by region (NUTS 2) in 2457 dogs

Region (NUTS 2)	*n* of dogs tested (%)	% of seropositive dogs^a^ (*n*)	95% CI
North	1638 (66.7)	23.0 (376)	20.9–25.1
Centre	592 (24.1)	33.8 (200)	30.0–37.8
Algarve	148 (6.0)	48.0 (71)	39.7–56.3
Autonomous Region of Madeira	44 (1.8)	15.9 (7)	6.6–30.1
Alentejo	20 (0.8)	35.0 (7)	15.4–59.2
Autonomous Region of the Azores	9 (0.4)	33.3 (3)	7.5–70.1
West and Tagus Valley	1 (0.0)	0.0 (0)	0.0–95.0
Setúbal Peninsula	5 (0.2)	0.0 (0)	0.0–36.9
Total	2457 (100)	27.0 (664)	25.3–28.8

*CI* confidence interval, *NUTS 2* Nomenclature of Territorial Units for Statistics

^a^*χ*^2^ = 66.2, *df* = 7, *P* < 0.001

**Figure 2:**
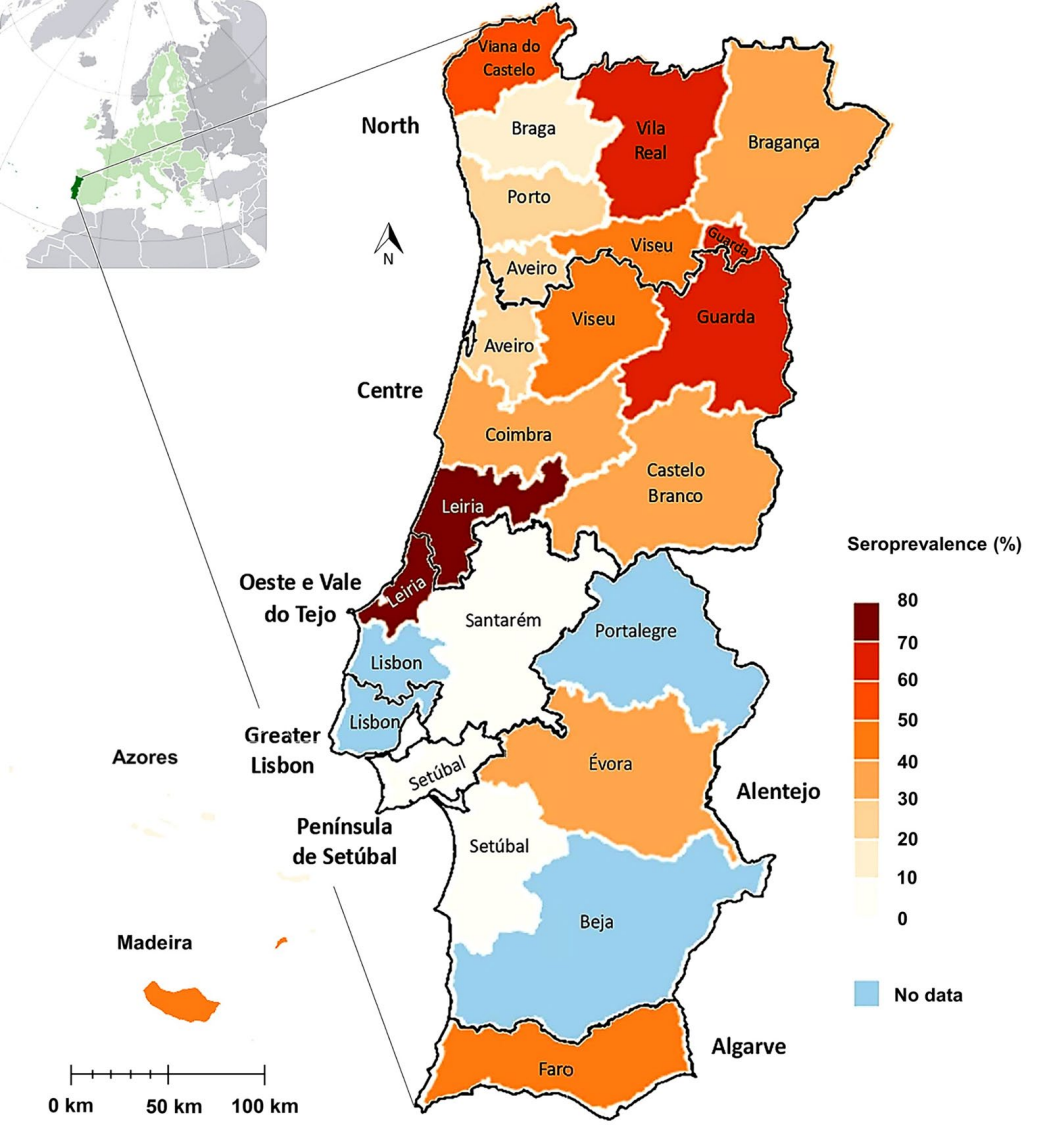
Map of Portugal showing categorical representation of seroprevalence over 12 years (2013–2024) for *Rickettsia conorii* seroprevalence determined by IFAT per district and NUTS 2 (map drawn in: www.mapinseconds.com; accessed on 21 January 2025). Prevalence values in districts with very low sample sizes (*n* ≤ 5) are not statistically robust and should be interpreted with caution. Uneven sample distribution may also reflect regional differences in clinical suspicion or awareness of *R. conorii*. *IFAT* immunofluorescence antibody test, *NUTS 2* Nomenclature of Territorial Units for Statistics

Dogs from the Algarve region had a 3.0 times higher risk (OR 3.0, 95% CI 1.7–5.4, *P* < 0.001) of presenting high positive titres (≥ 512) compared with the reference region, whilst those from the Centre region had a 2.3 times higher risk (OR 2.3, 95% CI 1.6–3.4, *P* < 0.001). Figure 2 displays seroprevalence over a 12-year period. The seroprevalence of *R. conorii* from the years 2013–2024 across the NUTS 2 regions is presented in Table 3. In addition, Tables 4 and 5 present the seroprevalence of *R. conorii* across regions in Portugal by season and month, respectively. No statistical association was observed between seropositivity to *R. conorii* and season (*χ*^2^ = 7.1, *df* = 3, *P* = 0.068) nor between seropositivity and month (*χ*^2^ = 12.7, *df* = 11, *P* = 0.313).

**Table 3:** Seroprevalence of *Rickettsia conorii* within each region (NUTS 2) across mainland Portugal and insular autonomous regions (2013–2024)

Evolution of the seroprevalence of *R. conorii* over 12 years													
Region	2013	2014	2015	2016	2017	2018	2019	2020	2021	2022	2023	2024	Average (%)
North	0.0%	0.0%	39.1%	42.3%	39.8%	36.0%	24.2%	22.0%	17.7%	12.9%	15.5%	19.1%	22.4
Centre	0.0%	NA	28.6%	50.0%	55.2%	36.4%	41.7%	30.1%	27.2%	31.9%	31.5%	40.7%	33.9
Algarve	NA	18.2%	0.0%	79.2%	54.2%	61.3%	68.8%	28.6%	28.6%	11.1%	9.1%	25.0%	34.9
ARM	NA	NA	0.0%	13.3%	20.0%	0.0%	0.0%	33.3%	40.0%	0.0%	0.0%	NA	11.9
Alentejo	NA	NA	NA	NA	NA	25.0%	50.0%	NA	0.0%	33.3%	100%	0.0%	34.7
ARA	NA	NA	NA	100%	NA	0.00%	NA	100%	0.0%	NA	NA	0.0%	40.0
PdS	NA	NA	NA	NA	0.0%	NA	NA	0.0%	NA	0.0%	NA	NA	0.0
OVT	NA	NA	NA	NA	NA	NA	NA	NA	0.0%	NA	NA	NA	0.0
Average	0.0%	9.1%	16.9%	57.0%	33.8%	26.5%	36.9%	35.7%	16.2%	14.9%	31.2%	17.0%	22.2

*ARA* Autonomous Region of the Azores, *ARM* Autonomous Region of Madeira, *OVT* West and Tagus Valley, *PdS* Setúbal Peninsula, *NA* not available, *NUTS 2* Nomenclature of Territorial Units for Statistics

**Table 4:** Seroprevalence of *Rickettsia conorii* within each region across Portugal by month in 2457 dogs

Month	Region (NUTS 2)						Total (%)^a^
	North	Centre	Algarve	ARM	Alentejo	ARA	
January	17	16	4	1	1	0	39 (5.9)
February	21	11	3	0	1	0	36 (5.4)
March	26	11	7	0	1	0	45 (6.8)
April	27	28	12	1	1	0	69 (10.4)
May	40	15	8	0	0	1	64 (9.6)
June	44	21	6	2	0	0	73 (11.0)
July	36	17	7	0	1	0	61 (9.2)
August	48	24	4	2	1	0	79 (11.9)
September	38	10	5	1	0	2	56 (8.4)
October	31	13	3	0	0	0	47 (7.1)
November	24	18	8	0	0	0	50 (7.53)
December	24	16	4	0	1	0	45 (6.8)
Total	376	200	71	7	7	3	664 (100)

*ARA* Autonomous Region of the Azores, *ARM* Autonomous Region of Madeira, *NUTS 2* Nomenclature of Territorial Units for Statistics

^a^*χ*^2^ = 12.7, *df* = 11, *P* = 0.313

**Table 5:** Seropositivity to *Rickettsia conorii* within season in 2457 dogs

Season	*n* of dogs tested (%)	% of seropositive dogs^a^ (*n*)	95% CI
Winter	623 (25.4)	26.5 (165)	22.9–30.4
Spring	692 (28.2)	29.8 (206)	26.5–33.3
Summer	711 (28.9)	27.6 (196)	24.5–30.9
Autumn	431 (17.5)	22.5 (97)	18.6–26.9
Total	2457 (100)	27.0 (664)	25.3–28.8

Winter, 21 December−19 March; spring, 20 March−20 June; summer, 21 June−21 September; autumn, 22 September−20 December. *CI* confidence interval

^a^*χ*^2^ = 7.1, *df* = 3, *P* = 0.068

### Size

For statistical analysis related to breed size, the mixed-breed category was not included, resulting in 1140 dogs across 88 breeds being considered (Table 6). The chi-squared test revealed a statistically significant difference amongst the five breed size categories (*χ*^2^ = 13.2, *df* = 4, *P* = 0.010). The logistic regression analysis indicated that giant breeds had significantly higher risk (OR 3.0, 95% CI 1.3–6.6, *P* = 0.008) of testing seropositive for *R. conorii* compared with the reference category (arbitrarily assumed to have an OR 1.0), that is, toy breeds (14.3%). The OR for small, medium and large breed groups were not statistically significant.

**Table 6:** Seropositivity to *Rickettsia conorii* by breed size in 1140 dogs

Titre		Breed size^a^				Total
	Toy	Small	Medium	Large	Giant	
	% of dogs (*n*)	% of dogs (*n*)	% of dogs (*n*)	% of dogs (*n*)	% of dogs (*n*)	*n* (%)
< 64	6.2 (54)	11.5 (100)	22.1 (192)	51.4 (447)	8.9 (77)	76.3 (870)
64	4.1 (6)	12.3 (18)	25.3 (37)	48.6 (71)	9.6 (14)	12.8 (146)
128	4.2 (2)	8.3 (4)	25.0 (12)	52.1 (25)	10.4 (5)	4.2 (48)
256	0.0 (0)	13.8 (4)	13.8 (4)	55.2 (16)	17.2 (5)	2.5 (29)
512	3.2 (1)	0.0 (0)	16.1 (5)	54.8 (17)	25.8 (8)	2.7 (31)
1024	0.0 (0)	0.0 (0)	21.4 (3)	42.9 (6)	35.7 (5)	1.2 (14)
2048	0.0 (0)	50.0 (1)	0.0 (0)	0.0 (0)	50.0 (1)	0.2 (2)
Seropositive^a^	14.3 (63)	21.3 (127)	24.1 (253)	23.2 (582)	33.0 (115)	23.7 (1140)

Sera were tested starting at a 1:64 dilution, with a titre of 64 defined as the threshold for seropositivity (titres are expressed as the reciprocal of the corresponding dilutions)

^a^*χ*^2^ = 13.2, *df* = 4, *P* = 0.010

### Breed

The sample included 1317 mixed-breed dogs (53.6%), 176 Labrador Retrievers (7.2%), 139 German Shepherds (5.7%), 50 Yorkshire Terriers (2.0%), 45 Pinschers (1.8%), 45 Portuguese Podengos (1.8%), 41 Beagles (1.7%), 37 French Bulldogs (1.5%), 36 Boxers (1.5%), 36 Golden Retrievers (1.5%) and animals from 79 other breeds. The mixed-breed dogs had a seropositivity value of 29.9%. Like for size, the mixed-breed category was excluded, resulting in 1140 dogs across 88 breeds being considered.

The chi-squared test revealed a statistically significant association between breed and seropositivity to *R. conorii* (*χ*^2^ = 136.7, *df* = 88, *P* = 0.001). In the logistic regression model, Cavalier King Charles Spaniel was used as the reference category (due to its low seropositivity, that is, 10%, together a representation in the sample regarded as sufficient for that purpose, that is, *n* = 10). The Irish Setter (3 seropositive; 75.0%) and Miniature Schnauzer (3 seropositive; 75.0%) had significantly higher risk of seropositivity (OR 27.0, 95% CI 1.3–578.4, *P* = 0.035) compared with the reference category. Similarly, the Portuguese Podengo (22 seropositive; 48.9%) revealed increased risk of seropositivity, that is, OR 8.6 (95% CI 1.0–73.7, *P* = 0.049). The OR of no other breed were statistically significantly different, showing that the observed variability in seropositivity was restricted to a few breeds.

### Sex

Table 7 represents the percentage of positive and negative to *R. conorii* according to sex. The difference observed between animal sexes was statistically significant (*χ*^2^ = 5.2, *df* = 1, *P* = 0.023), with males presenting the highest risk (OR 1.2, 95% CI 1.0–1.5, *P* = 0.023).

**Table 7:** Seropositivity to *Rickettsia conorii* by sex in 2457 dogs

Sex	*n* of dogs tested (%)	% of seropositive dogs (*n*)	95% CI
Female	1106 (45.0)	24.8^a^ (274)	22.2–27.6
Male	1351 (55.0)	28.9^a^ (390)	26.4–31.6
Total	2457 (100)	27.0 (664)	25.3–28.8

*CI* confidence interval

^a^χ^2^ = 5.2, *df* = 1, *P* = 0.023

### Age

From the 2457 dogs that were analysed, age data were available only for 2052 of them. In fact, for 405 dogs (16.5%) the requisition file did not specify age and they were thus excluded from this analysis. Age distribution amongst these 2052 dogs ranged from 6 months (≤ 1 year) to 20 years (≥ 11 years), with a median age of 5 years (interquartile range: 2–9 years). Table 8 displays seropositivity to *R. conorii* according to age group. The results of the chi-squared test analyses reveal association between age groups and *R. conorii* seropositivity (*χ*^2^ = 47.7, *df* = 4, *P* < 0.001). Puppies (< 1 year) had the lowest seropositivity value (9.2%, 95% CI 5.3–15.3) and served as the reference category (OR 1). Young dogs (1 to < 2 years) had a significantly higher risk of seropositivity (OR 2.3, 95% CI 1.2–4.4, *P* = 0.011). Adults (2 to < 6 years) also had a higher risk (OR 4.2, 95% CI 2.3–7.6, *P* < 0.001) and so had senior dogs (6 to < 11 years; OR 3.4, 95% CI 1.9–6.2, *P* < 0.001). Geriatric dogs (≥ 11 years) had the highest seropositivity value (Table 8) and associated risk (OR 5.2, 95% CI 2.8–9.7, *P* < 0.001).

**Table 8:** Seropositivity to *Rickettsia conorii* by age group in 2052 dogs

Age group (years)	*n* of dogs tested (%)	% of seropositive dogs^a^ (*n*)	95% CI
Puppy (< 1)	141 (6.8)	9.2 (13)	5.0–15.3
Young (1 to < 2)	263 (12.8)	19.0 (50)	14.5–24.3
Adult (2 to < 6)	644 (31.4)	29.8 (192)	26.3–33.5
Senior (6 to < 11)	709 (34.6)	25.8 (183)	22.6–29.2
Geriatric (≥ 11)	295 (14.4)	34.6 (102)	29.2–40.3
Total	2052 (100)	26.3 (540)	24.4–28.3

*CI* confidence interval

^a^*χ*^2^ = 47.7, *df* = 4, *P* < 0.001

## Discussion

The present study provides a detailed and comprehensive analysis of exposure to or infection with *R. conorii* in dogs across Portugal, offering valuable insights into the epidemiology of this vector borne agent. The findings reveal associations between seropositivity and several variables/categories, including geographical distribution, size, breed, sex and age.

In this study, 27.0% of the analysed dogs tested positive for *R. conorii*, with regional seroprevalence values ranging from 0.0% (West and Tagus Valley and Setúbal Peninsula) to 48.0% (Algarve). It is important to note that regions with 0.0% seroprevalence may reflect a limited number of animals tested or reduced clinical awareness, rather than a true absence of exposure, and should therefore be interpreted with caution. This pattern is consistent with previously reported variability in seropositivity across different regions of Portugal and other countries of the Mediterranean Basin [10, 38, 42, 43].

The considerably higher risk of seropositivity for high-positive titres (≥ 512) in dogs from the Algarve (OR 3.0) and Centre (OR 2.3) regions highlights regional differences in exposure to *R. conorii*. These findings are consistent with the known ecological and environmental conditions in southern Portugal, which include a warm and dry Mediterranean climate that supports high densities of *Rh. sanguineus* s.l., the primary vector of *R. conorii*. The elevated risk suggest ongoing transmission in these areas, likely due to persistent exposure to infected ticks [44, 45]. This pattern is supported by previous studies in Portugal and other countries in the Mediterranean Basin, such as Spain and Italy, which report similar trends in regions with comparable environment and vector habitats [46, 47].

In apparent contrast with the present data, a recent study focussing on northern Portugal reported a seroprevalence of 9.7% for *R. conorii* using a commercial IFAT test [10]. This lower seroprevalence may reflect regional ecological differences, variations in populations (e.g. shelter dogs versus clinically suspected dogs) or temporal fluctuations in seroprevalence. The variability in reported seroprevalence values emphasises the importance of considering factors such as geographical location, study population characteristics and temporal changes when interpreting *R. conorii* prevalence data in Portuguese dogs. For example, another study found that 38.5% of 400 healthy dogs in southern Portugal (the Algarve region) had IgG antibodies reactive with *R. conorii*, further highlighting regional and population-based differences in seropositivity values [38]. These observed patterns may be attributed to environmental variations, differences in vegetation and wildlife reservoir distribution, all of which influence the abundance and spread of *Rh. sanguineus* s.l. [48–52].

However, the present study did not find an association between seasons or months and *R. conorii* seropositivity. This contrasts with previous reports from Spain and France that pointed out seasonal trends in prevalence, typically linked to peak tick activity during warmer months [21, 53–55]. The absence of seasonality in our findings may indicate that exposure to or infection with *R. conorii* in dogs occurs throughout the year in Portugal, likely due to the Mediterranean climate, which supports a regular tick activity.

The relationship between breed size and susceptibility to *R. conorii* has not been thoroughly investigated thus far. The present study suggests that breed size plays a role in the variability of *R. conorii* seropositivity, with giant breeds being more exposed to or infected with this agent. One study in dogs from northwestern Spain points out that larger breeds, often used for guarding, herding or other outdoor activities, are more likely to be exposed to ticks due to their environmental interactions and roles, particularly in rural or semi-rural settings [53]. In the present study, giant breeds were found to have significantly higher risk of testing positive for *R. conorii* compared with toy breeds. This observation aligns with the understanding that dogs used for outdoor roles, such as guarding, hunting or herding, frequently inhabit environments with a higher tick abundance and density, a circumstance which increases their risk of tick infestation and potential exposure to and infection with *R. conorii*. Furthermore, rural settings, often associated with these roles, provide favourable conditions for tick populations, such as dense vegetation and the presence of wildlife reservoirs, which may contribute to increased transmission risks [20, 34, 43, 56, 57].

It is crucial to recognise that the observed association between breed size and seropositivity is unlikely to be solely attributable to size. Instead, it reflects a complex interplay of factors, including the dog’s activity, environmental exposure and behavioural tendencies. Large and giant breeds are more likely to spend prolonged periods outdoors, a circumstance which increases their interaction with tick habitats. However, the relationship between breed size and vector-borne disease susceptibility is complex and context dependent, influenced by factors such as specific pathogens involved, geographical location and local ecological conditions [58, 59]. Future research should aim at clarifying whether breed size directly influences susceptibility due to physiological or genetic predispositions, or whether the observed patterns are predominantly driven by behavioural and environmental factors. A deeper understanding of these dynamics could improve targeted preventative strategies, such as tailored tick control measures for larger breeds frequently exposed to high-risk environments.

The identification of the Irish Setter and Miniature Schnauzer breeds as having a higher risk of *R. conorii* seropositivity is a novel finding. To the present authors’ knowledge, breed-specific susceptibility has not been previously investigated, a circumstance which highlights the need for further research to explore potential genetic, behavioural and environmental factors that may influence infection with or exposure to *R. conorii* [10, 57, 60]. The current findings suggest that genetic factors may influence susceptibility, highlighting significant interbreed variability in *R. conorii* seroprevalence [34, 50].

The finding that males demonstrated a statistically different seroprevalence of *R. conorii* (28.9%) compared with females (24.8%) (Table 7) contrasts with previous studies from Portugal, Egypt and Brazil [10, 57, 61], which reported higher seroprevalence amongst females. This discrepancy might be explained by differences in the study populations, geographical settings or environmental factors influencing tick exposure. In the present study, males’ higher seroprevalence could be linked to behavioural factors, such as greater outdoor activity or exploration of environments conducive to tick infestations. In contrast, the higher risk observed in females in a previous study conducted in Portugal [10] may reflect specific environmental conditions, such as confined spaces, population density or altered exposure dynamics, as the study was conducted in shelter settings.

It is crucial to note that sex-based differences in susceptibility to vector-borne diseases are complex and may not solely result from behavioural or environmental factors. Intrinsic factors, such as hormonal influence or immune system variations, could also play a role, although such mechanisms remain underexplored in dogs and other animals exposed to or infected with *R. conorii* [20, 59, 62]. The present findings highlight the need for further investigation into the interplay between sex, behaviour and environmental exposure in determining seropositivity to *R. conorii*. Understanding these dynamics is essential for designing effective prevention strategies that address both intrinsic and extrinsic risk factors [63–65].

In this study, 26.3% of the dogs analysed for age tested positive for *R. conorii*, with seroprevalence increasing progressively from 9.2% in puppies (< 1 year; reference category) to 34.6% in geriatric dogs (≥ 11 years; OR 5.2) (Table 8). An association between age and seropositivity was observed, as all the age groups had a significant risk factor compared with the reference one, suggesting cumulative exposure to ticks over time. These findings align with limited existing literature indicating higher seroprevalence to tick-borne pathogens, including *Rickettsia* spp. values in older dogs [26, 57, 60, 66–69]. Nevertheless, the scarcity of studies addressing the relationship between age and *R. conorii* seropositivity highlights the need for further research to better understand the role of age in the epidemiology of vector-borne infections. It is also important to emphasise that seropositivity reflects past exposure and does not necessarily indicate current infection, particularly in the absence of clinical signs or molecular confirmation.

The associations identified between seropositivity and factors such as geographical region, size, breed, sex and age provide a comprehensive understanding of the determinants influencing seropositivity risk. Regional variability, with seroprevalence ranging from 0.0% to 48.0%, suggests the importance of geographical factors in shaping exposure to tick vectors. Notably, breed-specific differences, including the increased risk of seropositivity in giant breeds and the identification of Irish Setter and Miniature Schnauzer breeds as higher-risk breeds, suggest behavioural and environmental factors linked to outdoor activities may drive exposure. Similarly, the associations observed for sex and age suggest that cumulative environmental exposure and behavioural patterns contribute to the infection risk.

This study faced some limitations that must be recognized. All dogs were clinically suspected of a tick-borne disease on the basis of the medical evaluation performed in veterinary medical centres, which included signs such as fever and lethargy. This circumstance may have biased the true seroprevalence values. The lack of complete clinical data and the exclusive use of serology for antibodies to *R. conorii* have restricted the ability to confirm active infections. A recognised limitation of IFAT, despite its widespread use in seroepidemiological studies, is the potential for cross-reactivity with other *Rickettsia* spp. belonging to the SFG, which may result in seropositivity that is not specific *to R. conorii*. Whilst the commercial kit used in this study was based on *R. conorii* antigens, the potential for cross-reactions should be considered when interpreting results, particularly in regions where other SFG species may be circulating. Furthermore, the limited or absent data from certain regions (e.g. Greater Lisbon) hindered a more comprehensive epidemiological analysis. Future studies should aim at adopting a prospective design to enhance the standardisation of data collection, including information on clinical signs and clinicopathological abnormalities, co-infections and other comorbidities. Incorporating molecular diagnostic methods alongside serological testing will enable the confirmation of active infections and the identification of different species and even different strains. This is particularly relevant in cases where antibody titres are high (e.g. ≥ 512), as molecular techniques such as polymerase chain reaction (PCR) can help confirm current or recent infections and reduce the diagnostic uncertainty associated with serological cross-reactivity. Expanding sample collection to underrepresented regions and implementing longitudinal studies across different seasons will allow for a more robust analysis of spatial and temporal variations in prevalence [70].

## Conclusions

The findings of the present study illustrate the seroprevalence of *R. conorii* in dogs across Portugal, identifying significant associations with geographical region, size, breed, sex and age. Considering the limited research on this subject within the country, these data provide a contribution to the understanding of MSF in endemic regions. The study also highlights the importance of integrating regional epidemiological data with broader behavioural, environmental and genetic considerations. Such an approach, framed within a One Health perspective, is useful to the development of targeted surveillance systems and evidence-based preventive strategies, thereby advancing the management of *R. conorii* in both veterinary and public health contexts.

## Data Availability

Data supporting the conclusions of this article are included within the article. The datasets used and/or analysed during the present study are available from the corresponding author on reasonable request.
